# Subcellular Localization of the TFF Peptides xP1 and xP4 in the *Xenopus laevis* Gastric/Esophageal Mucosa: Different Secretion Modes Reflecting Diverse Protective Functions

**DOI:** 10.3390/ijms21030761

**Published:** 2020-01-23

**Authors:** Heinz Schwarz, Werner Hoffmann

**Affiliations:** 1 Max-Planck-Institute for Developmental Biology, Max-Planck-Ring 5, 72076 Tübingen, Germany; heinz.schwarz@tuebingen.mpg.de; 2 Institute of Molecular Biology and Medicinal Chemistry, Otto-von-Guericke University Magdeburg, Leipziger Str. 44, 39120 Magdeburg, Germany

**Keywords:** electron microscopy, trefoil factor, TFF1, TFF2, goblet cell, lectin, mucin granules, secretion, secretory granules, stomach

## Abstract

The TFF peptides xP1 and xP4 from *Xenopus laevis* are orthologs of TFF1 and TFF2, respectively. xP1 is secreted as a monomer from gastric surface mucous cells and is generally not associated with mucins, whereas xP4 is a typical secretory peptide from esophageal goblet cells, and gastric mucous neck and antral gland cells tightly associated as a lectin with the ortholog of mucin MUC6. Both TFF peptides have diverse protective functions, xP1 as a scavenger for reactive oxygen species preventing oxidative damage and xP4 as a constituent of the water-insoluble adherent inner mucus barrier. Here, we present localization studies using immunofluorescence and immunoelectron microscopy. xP1 is concentrated in dense cores of secretory granules of surface mucous cells, whereas xP4 mixes with MUC6 in esophageal goblet cells. Of note, we observe two different types of goblet cells, which differ in their xP4 synthesis, and this is even visible morphologically at the electron microscopic level. xP4-negative granules are recognized by their halo, which is probably the result of shrinkage during the processing of samples for electron microscopy. Probably, the tight lectin binding of xP4 and MUC6 creates a crosslinked mucous network forming a stabile granule matrix, which prevents shrinkage.

## 1. Introduction

The secretory peptides xP1 and xP4 belong to the family of trefoil factor family (TFF) peptides and represent the *Xenopus laevis* orthologs of mammalian TFF1 and TFF2, respectively [1,2,3]. As a hallmark, TFF peptides contain a cysteine-rich module containing six disulfide-linked cysteine residues [4]. Generally, this peptide family plays a role in the protection of mucosal epithelia [4,5,6]. xP1 and xP4 are differentially expressed in the gastrointestinal tract of *X. laevis* [7]. xP1 is synthesized in gastric surface mucous cells [1,7], whereas xP4 is released from esophageal goblet cells as well as gastric mucous neck and antral gland cells [1,7]. Of note, a xP1 homolog, which has been termed xP1-L, is expressed in the stomach of *X. laevis* in larval stages and tadpoles, but not in adults [8].

xP4 occurs in two variants, a N-glycosylated form (xP4.1) and a non-glycosylated form (xP4.2). xP4.1 and xP4.2 are encoded by two genes characteristically differing in the sequence encoding the N-glycosylation site, which is lost in xP4.2 [9]. The existence of two xP4 genes is the result of a combination of two diploid progenitor species forming an allotetraploid species about 17–18 million years ago [10]. Interestingly, the subgenomes evolved asymmetrically [10]. This might be the reason why the expression of xP4.1 and xP4.2 differs characteristically: xP4.1 is synthesized with an increasing gradient from the gastric fundus to the antrum and weakly persists even into the anterior part of the intestine, but is not expressed in the esophagus [3,7,9]. In contrast, xP4.2 is expressed in the esophagus with a decreasing gradient from the gastric fundus to the antrum [3,7,9].

Recent data indicate that the molecular function of xP1 and xP4 is quite different [11]. xP1 contains a single TFF domain and an odd number of cysteine residues. It is secreted in an unusual monomeric form with an unpaired, activated cysteine residue [11]. The latter has been postulated to act as an extracellular scavenger for reactive oxygen/nitrogen species (ROS/RNS) [11]. This hypothesis would explain why *Tff1*-deficient (*Tff1^KO^*) mice obligatorily develop adenoma in the antrum, with about 30% progressing to carcinomas [12,13]. Furthermore, the majority of xP1 is not associated with mucins [11].

In contrast, xP4 contains four TFF domains and is exclusively associated with mucins [11]. Here, binding occurs to the *X. laevis* ortholog of mammalian MUC6 due to the lectin activity of xP4 [11]. As shown for pig and human TFF2, lectin binding to MUC6 is Ca^2+^-dependent and specific for the GlcNAcα1→4Galβ1→R moiety [14,15,16]. The terminal αGlcNAc at the non-reducing terminal is unusual and is recognized by the monoclonal antibody HIK1083 [17] and the lectin GSA-II from *Griffonia simplicifolia* [18]. This sugar epitope is conserved from *X. laevis* to humans [18], and even porcine TFF2 is able to bind to *X. laevis* gastric mucin [11]. The key enzyme for the synthesis of this terminal αGlcNAc residue is α1,4-N-acetylglucosaminyltransferase (α4GnT) [19]. Generally, co-expression of the lectin xP4/TFF2 and the carbohydrate GlcNAcα1→4Galβ1→R moiety is characteristic of gastric glands and appeared phylogenetically first in amphibians, when mucous neck cells developed [18]. The GlcNAcα1→4Galβ1→R moiety is specifically bound to the mucin MUC6, which is first detectable in frogs but is lost in teleost fish [20]. This evolutionary old lectin/mucin pair is typical of gastric and duodenal Brunner glands and is part of the firmly adherent, water-insoluble (inner) layer of the gastric mucus [15]. Thus, TFF2 stabilizes this mucus barrier, and this explains why *Tff2^KO^* mice showed accelerated progression to dysplasia after infection with *Helicobacter pylori* [21].

By complementing our structural and biochemical studies [1,7,9,11], we localize here xP1 and xP4 in the *X. laevis* esophagus and gastric mucosa, respectively, by the use of immunofluorescence and immunoelectron microscopy. As a prerequisite for such studies, strong and highly specific antisera against xP1 and xP4 were available [1,7]. The aim was to check whether there are differences between these two peptides, which are structurally related but have different protective functions [11]. This is a further step in order to gain knowledge in particular concerning the secretion of TFF peptides and their co-secreted mucins.

## 2. Results

### 2.1. Localization of xP1 in the X. laevis Gastric Mucosa

**Figure 1:**
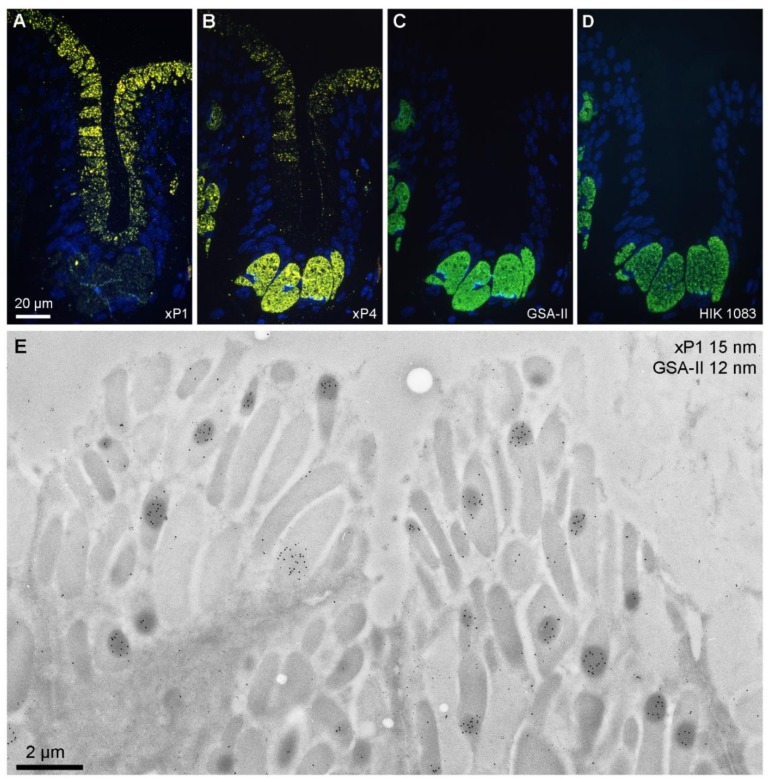
Labeled ultrathin methacrylate (Lowicryl K11M) sections of *X. laevis* stomach fundus. Fluorescence microscopy of sequential serial sections labeled for: **(A)** xP1 with antiserum anti-xP1-1 (Cy3, yellow); **(B)** xP4 with antiserum anti-xP4-1 (Cy3, yellow); **(C)** mucin with GSA-II (FITC, green); **(D)** mucin with antiserum HIK1083 (FITC, green). DNA of the cell nuclei was stained with DAPI (blue). Scale bar: 20 µm. **(E)** Electron micrograph of gold labeling with the anti-xP1-1 antiserum (detected with protein A-15 nm gold particles) and mucin staining with GSA-II (12 nm gold conjugates); scale bar: 2 µm.

As a first step, xP1 and, as controls, also xP4 as well as the *X. laevis* ortholog of mucin MUC6 were localized in the gastric mucosa using fluorescence microscopy on serial ultrathin sections (Figure 1A–D). xP1 (yellow) was localized in surface mucous cells (Figure 1A). In contrast, xP4 (yellow) was distributed mainly within the predominant mucous neck cells and minimally in surface mucous cells (Figure 1B). The MUC6 ortholog was exclusively localized within the mucous neck cells, either with the help of the lectin GSA-II (Figure 1C) or the antibody HIK1083 (Figure 1D).

Then, by the use of electron microscopy and immunogold labeling, xP1 (15 nm gold particles) and GSA-II reactivity (12 nm gold particles) were localized in the surface mucous cells of the gastric fundus (Figure 1E). As a hallmark, xP1 appeared mainly concentrated in electron-dense regions within specific secretory granules. GSA-II binding, as expected, was hardly detectable.

### 2.2. Localization of xP4 in the X. laevis Esophagus

**Figure 2:**
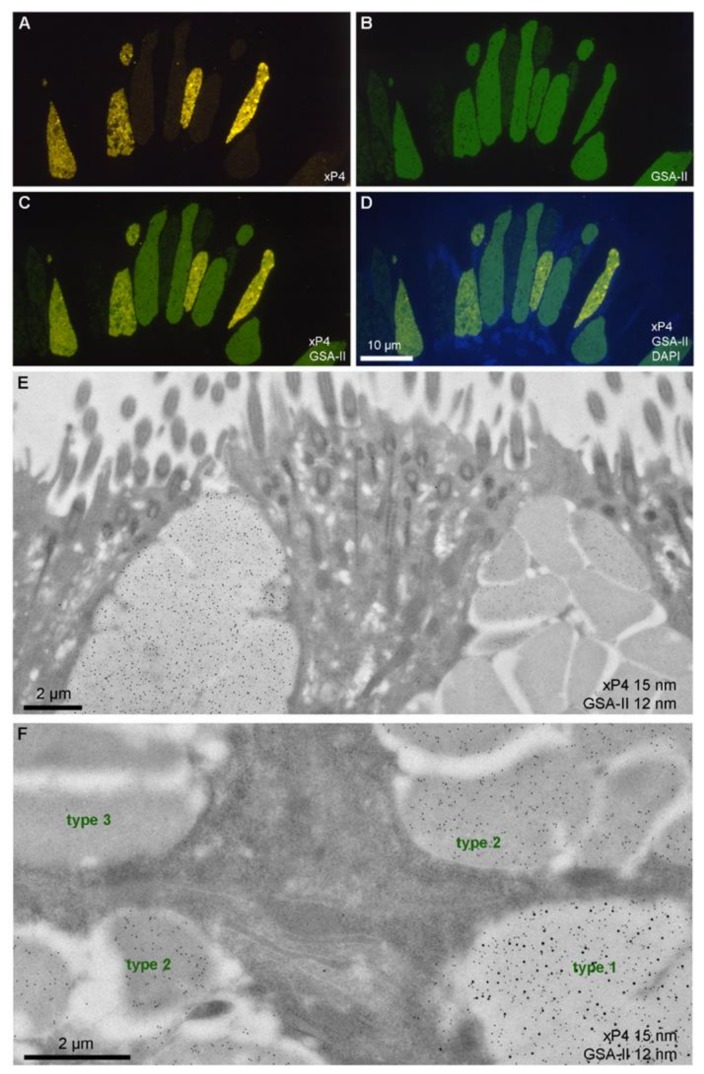
Labeled ultrathin methacrylate (Lowicryl K11M) sections of *X. laevis* esophagus. **(A–D)** Fluorescence microscopy of a single section labeled for xP4 with antiserum anti-xP4-1 (Cy3, yellow), mucin with GSA-II (FITC, green), and DNA of cell nuclei with DAPI (blue): xP4 (**A**), GSA-II (**B**), xP4/GSA-II double exposure (**C**), and xP4/GSA-II/DAPI triple exposure (**D**); scale bar: 10 µm. **(E, F)** Electron micrographs of gold labeling with the anti-xP4-1 antiserum (detected with protein A-15 nm gold particles) and mucin staining with GSA-II (12 nm gold conjugates) at two orthogonal orientations of the sectioning planes. The three types of secretory granules are marked in (**F**); scale bars: 2 µm.

As an initial step, xP4 and the *X. laevis* ortholog of mucin MUC6 were localized in the esophagus using fluorescence microscopy on the same section (Figure 2A–D). Clearly, labeling of xP4 (yellow) and the mucin MUC6 (detection with the lectin GSA-II; green) marked three different populations of goblet cells: (i) goblet cells positive for GSA-II and xP4; (ii) goblet cells positive for GSA-II, but nearly devoid of xP4; (iii) goblet cells hardly positive for GSA-II and lacking xP4.

In the next step, using electron microscopy and immunogold labeling, xP4 (15 nm gold particles) and MUC6 (detection with the lectin GSA-II; 12 nm gold particles) were localized at an ultrastructural level (Figure 2E,F). Each goblet cell contained multiple, relatively large granules (Figure 2E). There were two different types of granules observed: (i) granules with closely attaching borders, which nearly appear uniform (Figure 2E; goblet cell on the left side); (ii) granules with a white halo so that the individual granules appear separated (Figure 2E; goblet cell on the right side). Generally, the goblet cells were either of the “halo type” or the “uniform type”. We did not detect mixed types. 

The closely attaching granules were positive for both GSA-II and xP4 (GSA-II^POS^/xP4^POS^). As a hallmark, signals for both GSA-II and xP4 were evenly distributed within a granule. In contrast, the granules surrounded by a halo were either positive for GSA-II but not for xP4 (GSA-II^POS^/xP4^NEG^) or were negative for both GSA-II and xP4 (GSA-II^NEG^/xP4^NEG^). In Figure 2F, four neighboring goblet cells are shown in higher magnification, which contain the three different types of granules observed, i.e., GSA-II^POS^/xP4^POS^ (designated as type 1 granules), GSA-II^POS^/xP4^NEG^ (designated as type 2 granules), and GSA-II^NEG^/xP4^NEG^ (designated as type 3 granules). 

## 3. Discussion

### 3.1. Secretion of xP1 in Gastric Surface Mucous Cells: Concentration in Dense Core Regions of Secretory Granules

Using immunofluorescence microscopy, xP1 was exclusively detected in secretory granules of gastric surface mucous cells (Figure 1A). This is comparable with the localization of the mammalian ortholog TFF1 in humans and mice [22,23]. Also, the localization of xP4 mainly in mucous neck cells and little in surface mucous cells (Figure 1B) is similar to the mammalian ortholog TFF2 as shown in humans, mice, and rats [21,24,25]. However, in mammals, the localization in surface mucous cells is not that clear. In humans, aberrant expression of MUC6 in surface mucous cells has been reported in patients infected with *H. pylori* [26]. The exclusive staining of secretory granules of mucous neck cells with the lectin GSA-II as well as the antibody HIK1083 (Figure 1C,D) is indicative of the presence of the well-conserved terminal αGlcNAc residue in the carbohydrate moiety in the *X. laevis* ortholog of the mucin MUC6, which is an essential part of the recognition sequence for the lectin TFF2 and probably also xP4 [11,14,15].

At the electron microscopic level, xP1 was predominantly detectable in electron-dense cores of large secretory granules (Figure 1E). Thus, the TFF peptide xP1 is highly concentrated and does not appear to be mixed with the mucins in these cells. This is a major difference to the localization of xP4 in esophageal goblet cells (Figure 2E). However, the results are in full agreement with biochemical studies, which clearly showed that the vast majority of xP1 (about 97%) is not associated with mucin [11].

The localization of xP1 in dense core granules is comparable with that of TFF1 in murine gastric surface mucous cells, which also secrete the mucin Muc5ac [27]. In the past, the existence of granules with dense cores has been reported to occur in a variety of mucin secreting cells [28]. It is tempting to speculate that these dense cores mainly consist of highly concentrated xP1, which might even be able to form oligomers. In particular, the relatively low pH of these granules [29] would also allow association of the acidic C-terminal of xP1. The dense cores are surrounded by a mucin, which generally also serves as a gel matrix for mucous granules [29]. The intraluminal organization has been particularly investigated for MUC5AC [30,31]. Currently, the nature of the neutral mucin synthesized in high amounts in *X. laevis* surface mucous cells [7] is not known because at least in the closely related *Xenopus tropicalis*, no ortholog of the mammalian mucin MUC5AC was detected [20]. Generally, the formation of dense cores might occur already in the endoplasmic reticulum because here protein diffusion is about 100-fold faster than in mucin granules [30]. The reason for the different immobilization of the mucin matrix is the acidic intragranular pH [30].

### 3.2. Secretion of xP4 in Esophageal Goblet Cells: Different Types of Secretory Granules and Goblet Cells

From immunofluorescence microscopy, the goblet populations can be mainly distinguished by their ability to synthesize xP4 (Figure 2A). On the one hand, there are goblet cells present strongly staining for xP4 (Figure 2A). On the other hand, there are populations where xP4 is hardly or not detectable (Figure 2C). Currently, it is not clear whether these populations represent different, specialized goblet cell lines, which cannot interconvert. An argument favoring the hypothesis of relatively stable cell lines would be that the content in xP4 varies drastically in different granules within the same xP4-positive cell (Figure 2A,D). This is a sign that the expression rate of xP4 is highly regulated temporally in specific cells (xP4-positive cells) but obviously not in other cells (xP4-negative cells). Such a set-up would allow modulation of the mucus synthesis, where the rheological properties can be altered by a variable xP4 content according to the physiological needs.

These observations are in agreement with the results from electron microscopy. Here, goblet cells can be classified based on their types of granules. The goblet cells of the uniform type contain both xP4 and GSA-II positive mucin (Figure 2E,F). In contrast, in the goblet cells of the halo type, mainly a combination of type 2 and type 3 granules is present, i.e., they are mainly devoid of xP4 but contain variable amounts of GSA-II positive mucin (Figure 2E,F).

Taken together, Table 1 represents a classification of the goblet cell populations in *X. laevis* esophagus based on the criteria given above.

Furthermore, the question arises as to the the reason for the morphologically different appearance of the two goblet cell populations in the esophagus after electron microscopy (uniform- versus halo-type). One explanation would be that the halo-type cells got their appearance as a result of a shrinking process, which occurred during the dehydration processing of the samples for electron microscopy. Due to the relatively harsh processing conditions, specifically the secretory granules of the halo-type goblet cells could have been shrunk because of the lower matrix density of these granules. We hypothesize that the missing xP4 peptide is the reason for this shrinking process. It has been clearly shown in humans, pigs and *X. laevis* that TFF2 and its ortholog xP4, respectively, strongly associate with the mucin MUC6 via lectin binding [11,14,15]. This stabilizes the inner water-insoluble gastric mucus barrier physically in vivo [15]. For example, *Tff2^KO^* animals show accelerated progression of *Helicobacter*-induced gastritis to dysplasia particularly in the antrum because this is the preferred region for bacterial colonization [21]. Furthermore, TFF2 has been reported to affect the viscoelastic properties of mucous gels [32]. Thus, the presence of xP4 in mucous granules would be perfectly designed to prevent shrinkage during sample processing due to crosslinking and stabilization of the mucus matrix. This would explain why xP4 is a characteristic constituent of the granules of uniform-type goblet cells.

In addition, the even distribution of GSA-II-positive mucin and xP4 in the type 1 granules is a sign that the mucin and xP4 are mixed together. This is in line with the observation that xP4 is mucin-associated even after separation by size exclusion chromatography because of a non-covalent but strong lectin interaction [11]. The known high [Ca^2+^] in mucous granules [29] would be perfectly designed to facilitate the Ca^2+^-dependent lectin binding of TFF2/xP4 to MUC6 [14,15].

## 4. Materials and Methods

### 4.1. Preparation of Tissue Samples for Immunofluorescence and Immunoelectron Microscopy

The stomach, including the esophagus, was prepared from *X. laevis* (purchased from the Institute for Developmental Biology, Hamburg, Germany), immediately immersion-fixed in 4% formaldehyde, 50 mM HEPES, 1 mM CaCl_2_ on ice (0 °C), and shipped on ice (0 °C) to Tübingen for further processing. Tissue blocks were dehydrated in a series of ethanol baths at progressively lower temperatures according to the PLT method [33], and then embedded in Lowicryl K11M followed by UV polymerization at −35 °C. Sequential ultrathin sections of 50–70 nm thickness were mounted for subsequent affinity labeling either on polylysine-coated glass coverslips or EM grids.

All further technical details for immunofluorescence microscopy and immunoelectron microscopy have been described in detail previously [34,35]. In brief, immunofluorescence microscopy sections were incubated with the primary polyclonal antisera anti-xP1-1 [7] and anti-xP4-1 [1] both diluted 1:50, respectively, the monoclonal antibody HIK1083 (diluted 1:100), and the biotinylated lectin GSA-II (20 µg/mL) simultaneously. Detection of the antisera was with goat anti-rabbit IgG-Cy3 (diluted 1:500) and with goat anti-mouse IgG-FITC (diluted 1:300), whereas detection of biotinylated GSA-II was with mouse anti-biotin monoclonal antibodies (diluted 1:500) as a bridging antibody followed by goat anti-mouse IgG-FITC (diluted 1:300). DNA was counterstained with DAPI (0.1–0.4 µg/mL in H_2_O), and the sections were mounted in a Mowiol 4.88/glycerol mixture for semi-permanent embedding. Cy3 was imaged using a dedicated Cy3 filter (exciter HQ 545/30, dichroic mirror Q 570 LP and emitter HQ 610/75; F41-007, Chroma Brattleboro, VT, USA; delivered by AHF analysentechnik AG, Tübingen, Germany) resulting in a yellow signal, an FITC filter (HQ 480/40, Q 505 LP, HQ 535/50; F41-001), and a DAPI filter (D360/50, 400 DCP, GG 420 LP; F11-000).

In brief, for immunoelectron microscopy with post-embedding staining, unspecific binding sites were blocked with 0.5% bovine serum albumin, 0.2% gelatin in PBS, then the sections were treated with the primary polyclonal antisera anti-xP1-1 [7] and anti-xP4-1 [1], respectively, detected with protein A-15 nm gold particles (homemade, diluted 1:500) and fixed for 5 min with 4% formaldehyde in PBS. Then biotinylated lectin GSA-II (Vector B-1215, 20 µg/mL) was applied, which was detected with a monoclonal mouse anti-biotin antibody ( MCA 927 from AbD Serotec, now Bio-Rad AbD Serotec GmbH, Puchheim, Germany; diluted 1:500) followed by goat anti-mouse IgG-12 nm gold (Jackson 115-205-145, diluted 1:20). In general, all sera and conjugates were diluted in blocking buffer (0.5% bovine serum albumin, 0.2% gelatin in PBS) for fair comparison.

### 4.2. Lectins and Antisera

The following primary antisera and lectins were used: polyclonal rabbit antiserum anti-xP1-1 against the peptide FYPRATPEC [7]; polyclonal rabbit antiserum anti-xP4-1 against the peptide CFYPDIEDVTIIE [1] recognizing both glycoforms xP4.1 and xP4.2 [11]; biotinylated lectin GSA-II from *G. simplicifolia* (B-1215 from Vectorlabs, Burlingame, CA, USA). The mouse monoclonal antibody HIK1083 [17] was kindly provided by Prof. H. Ota.

Secondary antisera used for immunofluorescence microscopy: goat anti-rabbit IgG-Cy3 (Jackson 111-165-003, Jackson Immunoresearch, West Grove, PA, USA), mouse anti-biotin monoclonal antibodies (MCA927 from AbD Serotec; MB-9100 from Vectorlabs), goat anti-mouse IgG-FITC (Jackson 115-095-003).

Secondary antisera used for immunoelectron microscopy: home-made protein A-15 nm gold, mouse anti-biotin monoclonal antibodies (MCA927 from AbD Serotec; MB-9100 from Vectorlabs), goat anti-mouse IgG-12 nm gold (Jackson 115-205-146).

## 5. Conclusions

Taken together, the distribution of xP1 and xP4 in secretory granules of gastric surface mucous cells and esophageal goblet cells, respectively, is quite different. xP1 is highly concentrated in dense cores within the secretory granules, whereas xP4 appears evenly distributed within the granules together with the mucin MUC6. This is in line with recent biochemical studies [11], indicating a strong lectin-based association of xP4 with mucin, which stabilizes the gastric mucus to form a tight permeability barrier. In contrast, xP1 is not associated with mucin and predominantly occurs as a monomer with a reactive thiol group, which protects the gastric mucosa as a scavenger from reactive oxygen/nitrogen species.

## Figures and Tables

**Table 1:** Classification of goblet cell populations and secretory granules.

Goblet Cell	Granules	GSA-II/xP4
Uniform-type (xP4-positive)	Type 1	GSA-II^POS^/xP4^POS^
Halo-type (xP4-negative)	Type 2 Type 3	GSA-II^POS^/xP4^NEG^ GSA-II^NEG^/xP4^NEG^

## Abbreviations

DAPI	4’,6-diamidino-2-phenylindole dihydrochloride
TFF	Trefoil factor family
